# A Ring-Shaped Lateral Meniscus in an Anterior Cruciate Ligament Reconstruction Surgery

**DOI:** 10.7759/cureus.81729

**Published:** 2025-04-04

**Authors:** Mohd Fairudz Mohd Miswan, Sharifah Nor Amirah Alsagoff, Mohd Yusoff Yahaya, Mohamed Faizal Sikkandar, Mohd Shukry Mohd Khalid

**Affiliations:** 1 Sports Injury and Arthroscopy Unit, Department of Orthopaedics and Traumatology, Hospital Al-Sultan Abdullah, Puncak Alam, MYS; 2 Department of Orthopaedics and Traumatology, Faculty of Medicine, Universiti Teknologi MARA (UiTM), Sungai Buloh, MYS; 3 Department of Radiology, Hospital Al-Sultan Abdullah, Puncak Alam, MYS; 4 Department of Radiology, Faculty of Medicine, Universiti Teknologi MARA (UiTM), Sungai Buloh, MYS

**Keywords:** acl reconstruction, anatomical variant, discoid meniscus, lateral meniscus, ring-shaped meniscus

## Abstract

The ring-shaped meniscus is a rare variant of meniscus anomalies. We report a case of a young man who experienced right knee instability following a sports-related injury. Clinical examination suggested an anterior cruciate ligament (ACL) tear, which was confirmed through imaging studies. Intra-operatively, an incidental finding revealed the presence of a ring-shaped lateral meniscus. We discuss the diagnostic features of this uncommon abnormality.

## Introduction

The menisci in the knee joint are crescent-shaped structures made of fibrocartilage and are triangular in cross section. The medial meniscus is C-shaped, while the lateral meniscus has a more circular configuration. These structures are connected anteriorly by the transverse ligament and secured at the periphery by the coronary ligament. The menisci mainly consist of type I collagen and play a crucial role in deepening the articular surface.

Several morphological variations of the meniscus have been recognised, with discoid meniscus being the most prevalent anomaly, an abnormally thick and disc-shaped meniscus, covering a large area of the tibial plateau [1]. Other variants include the hypoplastic meniscus (small and underdeveloped), double-layered meniscus, and ring-shaped meniscus (RSM) [2]. The RSM is characterised by the presence of an intermeniscal bridge connecting the anterior and posterior horns giving the meniscus an appearance of a ring [3]. Its occurrence is relatively rare, with an incidence of up to 2.4% [4]. The Watanabe classification categorises lateral meniscus variants into three types: complete discoid, incomplete discoid, and Wrisberg ligament type [5]. Monllau et al. [6] proposed adding the RSM as a fourth variant within this classification due to its distinct morphology.

In this report, we present an incidental intra-operative finding of a lateral RSM, discovered during an anterior cruciate ligament (ACL) reconstruction surgery. Recognising the RSM is clinically important, as it can be mistaken for a meniscal tear on imaging or during surgery, potentially leading to unnecessary surgical interventions.

## Case presentation

**Figure 1:**
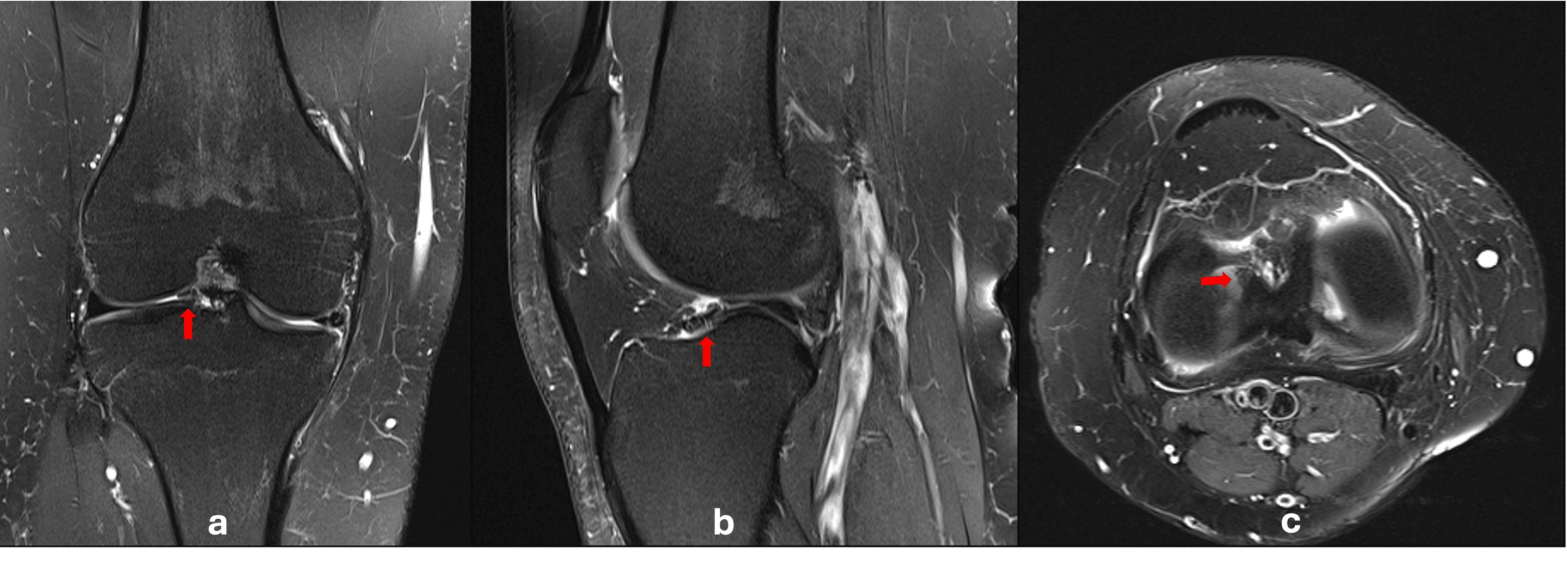
MR images showing the aberrant meniscal tissue (red arrow). (a) Coronal view demonstrating the inner portion of the ring-shaped meniscus, known as the "mirror image sign", a triangular structure mirroring the body of the meniscus. (b) Sagittal view showing "central bow tie sign" signifying a lateral meniscus fragment in the notch. (c) Axial view demonstrating a central portion or the intermeniscal bridge of the lateral meniscus fragment. MR: magnetic resonance

The patient is a 23-year-old male university student who plays competitive futsal and twisted his right knee in an attempt to avoid an opponent's tackle during a game. He heard a "pop" sound, fell to the ground, and noticed immediate swelling. He presented to our clinic three months after the injury with complaints of right knee instability despite having undergone physiotherapy. Physical examination revealed ACL deficiency where the anterior drawer and Lachman test were positive at grade 2. Other special tests were unremarkable. Magnetic resonance imaging (MRI) done showed an isolated high-grade partial ACL tear with the presence of meniscus-like tissue in the lateral compartment (Figure 1).

**Figure 2:**
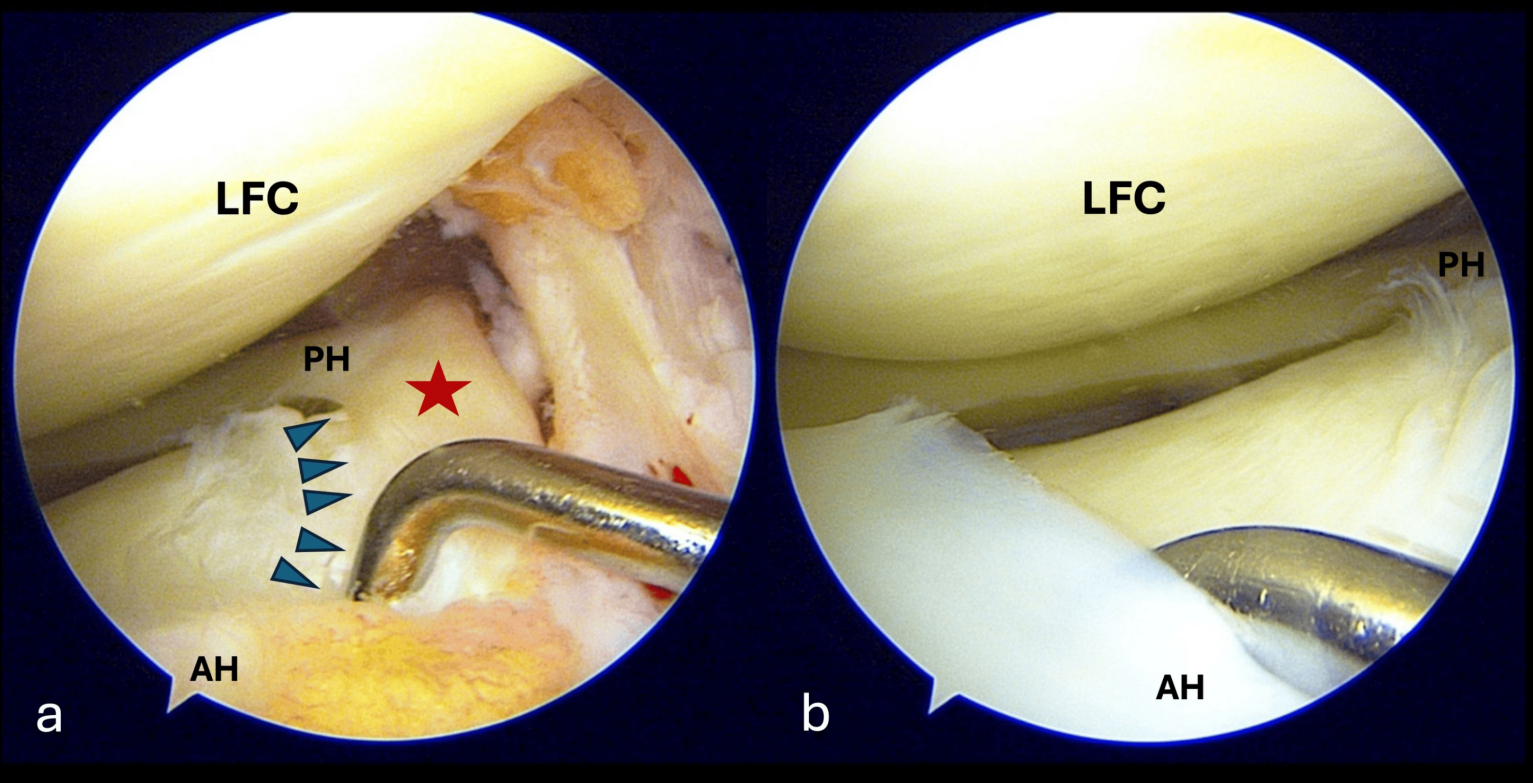
Arthroscopic findings of the lateral ring-shaped meniscus of the right knee (a) Intermeniscal bridge (red star) in between the anterior and posterior horns of the lateral meniscus and stable on probing. Blue arrowheads demarcating the inner edge of the intermeniscal bridge. (b) Anterior, lateral, and posterior aspects of the ring meniscus with no evidence of tears. AH: anterior horn; PH: posterior horn; LFC: lateral femoral condyle

**Figure 3:**
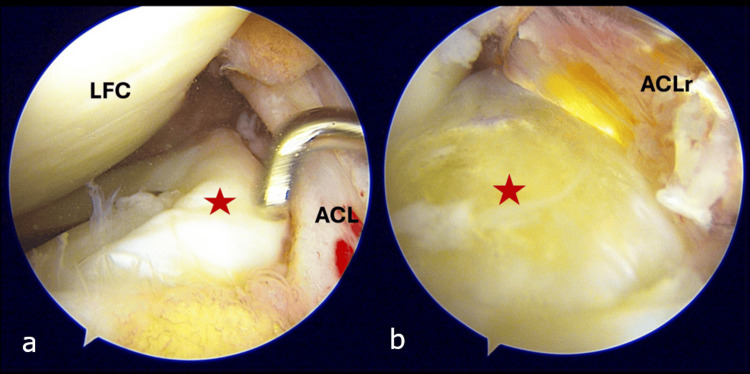
Arthroscopic image of the relation of the intermeniscal bridge to surrounding structures (a) Intermeniscal tissue firmly attached on the lateral tibial spine lateral to the ACL stump. (b) Relation between the inner portion of the RSM and the reconstructed ACL. ACL: anterior cruciate ligament; ACLr: reconstructed ACL; RSM: ring-shaped meniscus; LFC: lateral femoral condyle; red star: intermeniscal bridge of the lateral RSM

He was therefore scheduled for a right knee arthroscopic ACL reconstruction. During the arthroscopic evaluation, there was a near-complete ACL tear at the femoral origin. However, to our surprise, we also identified a ring-shaped morphology of the lateral meniscus. This was characterised by a meniscal tissue bridge connecting the anterior and posterior horns of the lateral meniscus (Figure 2a). Otherwise, there were no tears seen (Figure 2b). The structure was located on top of the lateral tibial spine and lateral to the ACL fibers (Figure 3a). It resembled a meniscus, exhibiting a thicker outer border attached to the tibial surface and a free inner border. Its colour, shape, and consistency were comparable to that of a normal meniscal tissue. It was firmly attached and not mobile on probing, and edges were smooth and not degenerated. There was no evidence of tears. Notably, it did not obstruct the placement of the tibial tunnel, and it did not cause any impingement during knee motion post-reconstruction (Figure 3b).

## Discussion

RSM is a rare variant abnormality. It was first reported by Watson-Jones in 1930 [5] involving the medial meniscus, discovered incidentally during an autopsy. The majority of cases are found incidentally as it does not typically produce symptoms. This asymptomatic nature is largely attributed to its firm attachment onto the tibia and surrounding structures [7,8]. However, symptoms may arise when there is a concurrent pathology of the meniscus such as a parameniscal cyst or a meniscal tear [9].

RSMs are widely regarded as a congenital malformation, as discoid meniscus is a common observation in primates [10]. However, there have been few reports hypothesizing that the formation of the intermeniscal bridge may also occur as a potential regenerative response following ACL reconstruction [11-13] or meniscectomies [14,15]. In our case, the RSM is likely a developmental anomaly as the patient had no prior surgical procedures.

RSM has also been reported to be associated with ACL agenesis and ACL hypoplasia, which are even rarer congenital abnormalities [16,17]. However, this was not observed in our case. The patient experienced instability following a twisting injury to the knee. Further clarification with the reporting radiologist confirmed that the size, location, and position were consistent with a typical torn ACL and did not suggest hypoplastic features. Intra-operatively, although the origin of the ACL at the femoral condyle was almost completely torn, the distal stump seemed to be of normal size and footprint.

Distinguishing RSM from other diagnoses on MRI can be a challenge and is therefore often overlooked or misdiagnosed. After identifying this anomaly intra-operatively, we revisited the MRI and observed several notable features of RSM described by Koukoulias and Papastergiou [9]. On coronal images, the "mirror image sign" was noted [9], where the intermeniscal bridge appeared as a triangular structure mirroring the body of the meniscus (Figure 1a). Furthermore, its location adjacent to the tubercle of the intercondylar eminence rather than within the intercondylar notch aids in differentiating it from a bucket handle tear. On sagittal views, the "central bow tie sign" was likewise evident (Figure 1b). This resembles the typical bow tie appearance of the meniscal body but signifies the presence of the intermeniscal bridge at the centre [9].

An RSM can mimic a bucket handle meniscus tear, an incomplete discoid meniscus, or a discoid with a central tear [18]. While a bucket handle tear is usually mobile and reducible with visible torn edges, an RSM, as in our case, is firmly attached and cannot be displaced with a probe (Figure 2a). A central tear of a discoid meniscus would display an irregular and degenerative inner margin which was not present in our case. The tissue bridge connecting the meniscus horns in our case showed a tapered inner edge of the pristine meniscus tissue (Figure 2a). This meniscus anomaly did not cause any difficulty or obstruction to reconstruct the ACL (Figure 3). There was no need to compromise on the position of the tibial tunnel, and there was no overcrowding or impingement post-reconstruction.

## Conclusions

This is our first experience detecting a ring-shaped lateral meniscus. Diagnosis can be made through a thorough assessment of the MR images for features of RSM and an arthroscopic probing of the meniscus to differentiate from other meniscal pathologies and anomalies. It is usually asymptomatic unless there is presence of a tear or cyst. The standard reconstruction procedure was not affected by the presence of this variant anomaly. Differentiation from other meniscal pathologies is important pre-operatively so as to prevent unwarranted surgical intervention.
